# Evaluation of Fast Sample Entropy Algorithms on FPGAs: From Performance to Energy Efficiency

**DOI:** 10.3390/e24091177

**Published:** 2022-08-23

**Authors:** Chao Chen, Bruno da Silva, Ruiqi Chen, Shun Li, Jianqing Li, Chengyu Liu

**Affiliations:** 1 School of Instrument Science and Engineering, Southeast University, Nanjing 210096, China; 2 Department of Electronics and Informatics (ETRO), Vrije Universiteit Brussel (VUB), 1050 Brussels, Belgium; 3 VeriMake Research, Nanjing Renmian Integrated Circuit Technology Co., Ltd., Nanjing 210096, China; 4 College of Physics and Information Engineering, Fuzhou University, Fuzhou 350108, China

**Keywords:** sample entropy, complexity, time series, reconfigurable computing, FPGAs, performance, power efficiency, electrocardiogram (ECG)

## Abstract

Entropy is one of the most fundamental notions for understanding complexity. Among all the methods to calculate the entropy, sample entropy (SampEn) is a practical and common method to estimate time-series complexity. Unfortunately, SampEn is a time-consuming method growing in quadratic times with the number of elements, which makes this method unviable when processing large data series. In this work, we evaluate hardware SampEn architectures to offload computation weight, using improved SampEn algorithms and exploiting reconfigurable technologies, such as field-programmable gate arrays (FPGAs), a reconfigurable technology well-known for its high performance and power efficiency. In addition to the fundamental disclosed straightforward SampEn (SF) calculation method, this study evaluates optimized strategies, such as bucket-assist (BA) SampEn and lightweight SampEn based on BubbleSort (BS-LW) and MergeSort (MS-LW) on an embedded CPU, a high-performance CPU and on an FPGA using simulated data and real-world electrocardiograms (ECG) as input data. Irregular storage space and memory access of enhanced algorithms is also studied and estimated in this work. These fast SampEn algorithms are evaluated and profiled using metrics such as execution time, resource use, power and energy consumption based on input data length. Finally, although the implementation of fast SampEn is not significantly faster than versions running on a high-performance CPU, FPGA implementations consume one or two orders of magnitude less energy than a high-performance CPU.

## 1. Introduction

Time series analysis in communication, physiological signal processing, financial analysis, and other applications makes extensive use of information entropy. Entropy can be used to assess market risk [1], where maximal entropy is used to pick portfolios and price assets [2]. Different methods such as approximate entropy (ApEn), sample entropy (SampEn), fuzzy entropy (FuzzyEn) and permutation entropy (PE) exist to extract the information entropy. Due to its simple definition, SampEn [3] is much more robust and occupies less computing burden than some more sophisticated entropy algorithms. SampEn is derived from ApEn, a useful method proposed in 1991 by Pincus [4]. SampEn reduces bias by avoiding the self-match in ApEn and is more consistent than ApEn. Moreover, SampEn also performs well with both short and lengthy data sets. Because of the advantages described above, SampEn has become a widely used entropy estimation algorithm.

SampEn is frequently employed in chaotic systems [5], climatic complexity [6], language recognition [7,8], industrial defect analysis [9] and especially in biological signal analysis [10,11,12,13]. For instance, SampEn is a promising and useful method for deducing atrial fibrillation (AF) from ECG data without intrusive testing. In ECG datasets, SampEn might be used to examine both atrial activity and ventricular activity [10].

During the ketogenic diet (KD) therapy, researchers used SampEn to evaluate 7680 (30 s) points EEG segments in patients with intractable pediatric epilepsy [14].

Unfortunately, SampEn still takes a long time and slows down actual performance in real-world applications due to its time-consuming computation. Short data lengths are often used in projects to reduce the computational burden on general computers, although accuracy suffers as a result. This limitation restricts SampEn’s real-time usage, while it can detect an abnormal spot in offline analysis. At the same time, many researchers explore to offload entropy algorithms on performance and power efficient technologies, such as field-programmable gate arrays (FPGAs). Moreover, FPGAs have been often used for fast signal processing, such as processing biomedical signals [15,16], but especially due to their capability of processing in pipeline. For instance, FPGAs have implemented entropy algorithms for applications such as failure detection in motors [17,18], chaotic systems [5,19], and seamless measurements of transient signals [20]. These works, however, have only built basic implementations of entropy algorithms. A hardware implementation on FPGAs of a straightforward version of SampEn [21,22] cannot be applicable due to numerous unnecessary comparisons, highly degrading the achieved performance.

The unnecessary similarity comparisons in straightforward (SF) defined SampEn represent a significant load. Simple SampEn is time consuming with O(n2) time complexity. Many new SampEn algorithms have been proposed and implemented during the last few years on the software platform [23,24,25,26,27,28]. Because they attain the same results as defined SampEn, fast SampEn of bucket-assisted (BA) and sort-based lightweight (LW) algorithms attract our attention. These algorithms save computing time by eliminating superfluous SampEn similarity comparisons and relieving the burden of simple SampEn. However, regardless of the quantity of data records or the length of the data, the computing overhead in big data remains high.

SampEn hardware implementations on FPGAs are efficient in computing, avoiding complicated software dependencies and relying only on underlying hardware resources for calculation. FPGA is a powerful hardware platform with reconfiguration ability [29] and benefits in speed and power efficiency [30,31,32]. Although the mentioned fast SampEn algorithms are proposed for general-purpose computational units (CPUs), they also promise higher performance while demanding lower power consumption when ported to FPGAs. In fact, the power efficiency of this technology would lead to suitable solutions to be integrated on embedded systems. To our knowledge, it is the first time that these fast SampEn algorithms are evaluated on FPGAs.

Our evaluation bridges the gap between hardware technologies and fast SampEn algorithms. The relevance of the input data length (number of elements) is here evaluated for fast SampEn algorithms on different hardware technologies, taking into consideration parameters such as the data series characteristics, the execution time, the hardware resource utilization, the power and energy consumption. Moreover, our analysis benefits the fast SampEn algorithms’ implementation, by unveiling their irregular memory access and the uneven intrinsic parallelism. Our analysis of the SampEn methods allows the extraction of design parameters of SampEn methods toward the release of the general processors burden by efficient hardware SampEn architectures.

In a summary, the main contributions of this work are as follows:

- Propose a broad methodology for designing fast SampEn hardware architectures for time series and validating them with Sine data and physiological ECG data.
- Quantify the uncertain storage space of fast SampEn algorithms using a universal framework that can be applied to a variety of data types.
- Provide computation latency estimations for fast SampEn implementations on FPGAs.
- Evaluate different fast SampEn algorithms on different computational technologies in terms of performance, resources, power and energy consumption.

The algorithms are described in Section 2. The methodology is described in Section 3. Section 4 deposits the key parameters in HW SampEn designs, performance estimation. The evaluation results of algorithms on CPUs and FPGAs are given in Section 5, and the results discussion is in Section 6. Finally, the conclusions are drawn in Section 7.

## 2. Algorithms

From the view of time complexity analysis for algorithms, SampEn is a time-consumptive $O(n^{2})$ complexity. Several algorithms have been proposed to hasten SampEn and decrease computation burden [24], reaching up to 10 times speedup compared to the original SampEn algorithm [26]. The optimized SampEn algorithms are based on some pre-ordering of the input values to accelerate the matching [24]. Due to the strong dependency of SampEn and the input data length, our analysis targets the impact of the data length *N* and how it affects the performance of the different implementations of SampEn.

Not all algorithms of fast SampEn are here considered because some fast SampEn algorithms ignore some similarity comparisons and lead to a different value with the defined SampEn [27,28]. The selected algorithms have high efficiency while maintaining the same stable SampEn value with concept-defined algorithms in both software and hardware experiments.

### 2.1. SampEn Definition

The SampEn algorithm checks the similarity of template vectors by making comparisons of dimensions *m* and m+1 of input data sequences. Then SampEn counts the matched number within tolerance *r* in the *m* scale and m+1 scale separately.

Suppose we have an N elements 1-D time series:

(1) $$x=x_{0},x_{1},x_{2},\ldots x_{N-2},x_{N-1}$$

A new template vectors series of scale *m* is constructed by series *x*. The vectors with *m* elements share same pattern with similar vectors. The ith template vector $X_{i}$ is constructed by

(2) $$X_{i}=x_{i},x_{i+1},\ldots ,x_{i+m-1}$$

The new vectors series are

(3) $$X=X_{0},X_{1},X_{2},\ldots X_{N-2},X_{N-m}$$

Two template vectors succeed in similarity match only when the Chebyshev distance (i.e., the maximum distance in elements, also known as maximum norm) is within the tolerance *r*:

(4) $$\left|\right|X_{i}-X_{j}{\left|\right|}_{m}=\left|\right|x_{i+k}-x_{j+k}\left|\right|<r,\;k=0,1,\ldots ,m-1,\;i\neq j\;$$

For all vectors, the total number of matched similarity results in *m* scale is called count1:

(5) $$count1=\sum_{i=0,j=0,i\neq j}^{N-1}\Theta \left(i,j,m,r\right)$$

where the similarity match result of two vectors is 0–1 function, and the result becomes 1 or 0 based on

(6) $$\Theta \left(i,j,m,r\right)\left\{\begin{array}{c} 1\;||X_{i}-X_{j}{\left|\right|}_{m}<r\\ 0\;\mathrm{otherwise} \end{array}\right.$$

For one template vector, the average probability of similarity match in *m* scale is called *B*:

(7) B=count1/(N−m+1)/(N−m)

Repeat the process in Formulas (2)–(6) by in the following scale of m+1.

The total number of similarity comparison results in m+1 scale is called count2:

(8) $$count2=\sum_{i=0,j=0,i\neq j}^{N-1}\Theta \left(i,j,m+1,r\right);$$

The average probability of similarity match in m+1 scale is called *A*:

(9) A=count2/(N−m)/(N−m−1)

SampEn is the negative logarithm of the conditional probability of the similarity match in *m* scale and m+1 scale (SampEn may have an undefined result in the condition of “no match”):

(10) $$\mathrm{SampEn}\left\{\begin{array}{c} \mathrm{Nan}\;\;\;\;A\;=\;0\\ -\ln(A/B)\;\; \end{array}\right.$$

### 2.2. Straightforward SampEn

An SF SampEn is described in Algorithm 1 by definition. SF SampEn directly compares the distances between all templates and then calculates the probability of similarity match at the *m* scale and m+1 scale. There are a large number of unnecessary matches in SF SampEn. In this study, we take the understanding that template vectors are similar in the m+1 scale only when they are matched at the *m* scale. Half matches can be reduced in this way. However, there are still many unnecessary similarity comparisons in SF SampEn, which explicitly failed in similarity matches and imposed a huge computational burden on the SampEn applications.
**Algorithm 1** Straightforward SampEn.  1:N: # elements, length of time sequence;  2:m: the dimension of template vectors;  3:$\;x=x_{0},x_{1},x_{2},\ldots x_{N-2},x_{N-1}$: time series;  4:$\;X^{m}=X_{0}^{m},X_{1}^{m},\ldots X_{N-m-1}^{m},X_{N-m}^{m}$: m scale template vector series  5:    where $X_{i}^{m}=\left(x_{i},...,x_{i-m+1}\right)$ is the $i_{th}$ vector of $X^{m}$;  6:count1=0;count2=0;  7:**for**i=0; i<N−1; i++**do**  8:      **for** j=0; j<N−1; j++ **do**  9:            **if** $abs(X_{i}^{m}-X_{j}^{m})\leq r$  **then**10:                  count1=count1+111:                  **if** $abs(x_{i+m}-x_{j+m})\leq r$  **then**12:                     count2=count2+113:                  **end if**14:            **end if**15:      **end for**16:**end for**17:count1=count1−N+m−1;    //extract self-match, otherwise for ApEn18:count2=count2−N+m;   //extract self-match, otherwise for ApEn19:B=count1/((N−m+1)∗(N−m));   //mean match probability in *m* scale20:A=count2/((N−m)∗(N−m−1));   //mean match probability in m+1 scale21:**if**A=0**then**22:      SampEn=Nan23:**else**24:      SampEn=log(B/A) //conditional probability and Entropy estimation25:**end if**26:return SampEn

### 2.3. Bucket-Assisted SampEn

The original BA algorithm has been proposed to accelerate approximate entropy (ApEn) in [26]. Due to the similarities of ApEn and SampEn, this algorithm has been proposed to further accelerate SampEn [24].

The BA SampEn algorithm described in Algorithm 2 uses buckets to screen high probability matched template vectors quickly. BA maps the index of template vectors into different buckets. If two template vectors are similar in *r*, their sums are still similar in m∗r. BA makes a new series by the sum of template vectors and makes extra buckets to store the candidate indexes. The number of buckets derives from the maximum and minimum value difference and their ratio to the threshold *r*. Then the new series is mapped to the corresponding bucket by their value. Potential similar templates will be in *m* adjacent buckets. In this way, template vectors do not need to compare all template spaces for similarity search, but only the adjacent bucket spaces, which can significantly reduce the comparison time. Here we utilize the basic BA algorithm by the understanding that template vectors in the *m* scale match at tolerance *r* only when the sum of them match within m×r.

**Algorithm 2** Bucket-assisted SampEn.
  1:N: # elements, length of time sequence;  2:m: the dimension of template vectors;  3:$\;x=x_{0},x_{1},x_{2},\ldots x_{N-2},x_{N-1}$: time series;  4:

$\;X=X_{0},X_{1},...X_{N-m-1},X_{N-m}$

  5:

$\;\;\;where\;X_{i}=\sum_{k=0}^{m-1}x_{i+k}$

  6:

$X_{min}=\min\left(X\right)$

  7:$X=X-X_{min}+1$ //correct *X* to avoid negative value  8:

$\;X_{max}=\max\left(X\right);$

  9:$\;N_{b}=\left\lceil X_{max}/r\right\rceil :$ # buckets10:

//bucketinitiation:

11:

count1=0;count2=0;

12:**for**i=0; i<N−1; i++**do**13:      $b=\left\lceil X_{i}/r\right\rceil$14:      $bucket_{b}=bucket_{b}\;⋃\;\left\{i\right\}$15:
**end for**
16:

//similaritysearch:

17:**for**$i_{b}=0$; $i_{b}<N_{b}$; $i_{b}++$**do**18:      $bound_{left}=\max\left(0,i_{b}-2\right)$19:      $bound_{right}=\min\left(N_{b},i_{b}+3\right)$20:      **for** $j_{b}=bound_{left}$; $j_{b}<bound_{right};j_{b}++$ **do**   //neighbour buckets21:            **for** $i\in bucket_{i_{b}}$ **do**22:                  **for** $j\in bucket_{j_{b}}$ **do**23:                        **if** $\left|\right|x_{index_{i}}-x_{index_{j}}{\left|\right|}_{m}<r$ **then**    //compare similarity in *m* scale24:                              count1=count1+125:                              **if** $\left|\right|x_{index_{i}}-x_{index_{j}}{\left|\right|}_{m+1}<r$ **then**   //compare similarity in m+1 scale26:                                     count2=count2+127:                               **end if**28:                         **end if**29:                   **end for**30:            **end for**31:      **end for**32:
**end for**
33:

//entropyestimate:

34:count1=count1−N+m−1;    //extract self-match, otherwise for ApEn35:count2=count2−N+m;    //extract self-match, otherwise for ApEn36:B=count1/((N−m+1)∗(N−m));    //mean match probability in *m* scale37:A=count2/((N−m)∗(N−m−1));    //mean match probability in m+1 scale38:
**if** A=0 **then**
39:     SampEn=Nan40:
**else**
41:     SampEn=log(B/A)    //conditional probability and Entropy estimation42:
**end if**
43:return SampEn


### 2.4. Lightweight SampEn

The LW algorithm is proposed in [24] to accelerate SampEn by pre-sorting the input sequence. However, the selected sorting algorithm is crucial in LW SampEn. Sorting algorithms in software have been well researched for their time complexity and space complexity. The most suitable sorting algorithm for FPGAs is also considered [33] in our analysis. The evaluated sorting algorithms are BubbleSort (BS) due to its simplicity and MergeSort (MS) due to its performance. MergeSort is a stable sorting algorithm whose worst and average time complexities are both O(NlnN). Additionally, in its hardware implementation on the FPGA, we use a bottom-up recursion to merge sequences from a small amount of data to a large amount of data. BubbleSort is selected for the sake of comparison. The evaluation of other sorting algorithms is out of the scope of this work.

LW SampEn described in Algorithm 3 sorts original sequences and stores their index. The sorting is based on the understanding that the matched vectors’ first element should also be in the distance within *r* tolerance. A sorted sequence can lead to a fast screen out of the potential matched vectors.
**Algorithm 3** Lightweight SampEn.  1:N: # elements, length of time sequence;  2:m: the dimension of template vectors;  3:$\;x=x_{0},x_{1},x_{2},\ldots x_{N-2},x_{N-1}$: time series;  4:y: sorted sequence of x and $y_{i}<y_{i+1}$  5:index: location in raw sequence, mapping element in *y* back to its location in *x*;  6:     where $y_{i}=x_{index_{i}}$  7:beginsortprosess:  8:sort time sequence and get sorted y and the index  9:endsortprocess10:count1=0;count2=0;11:**for**i=0; i<N−1; i++   **do**12:      **for** j=i+1; j<N; j++   **do**13:            **if** $y_{i}+r\leq y_{j}$ **then**14:                   **if** $index_{i},index_{j}<N-m+1$ **then**15:                         **if** $\left|\right|x_{index_{i}}-x_{index_{j}}{\left|\right|}_{m}<r$ **then**16:                               count1=count1+117:                               **if** $\left|\right|x_{index_{i}}-x_{index_{j}}{\left|\right|}_{m+1}<r$ **then**18:                                      count2=count2+119:                              **end if**20:                        **end if**21:                  **end if**22:            **end if**23:      **end for**24:**end for**25:B=count1/((N−m+1)∗(N−m));    //mean match probability in *m* scale26:A=count2/((N−m)∗(N−m−1));    //mean match probability in m+1 scale27:**if**A=0**then**28:    SampEn=Nan29:**else**30:    SampEn=log(B/A)     //conditional probability and Entropy estimation31:**end if**32:return SampEn

The time complexity of fast sorting is Nlog(N). The sorted sequence only needs to compare consistent template vectors within tolerance *r* in the sorted sequence, reducing the similarity search space. The sorted sequence, whose position is mapped to a template vector, could help judge whether the first element of the corresponding template vectors is matched between the tolerance *r* or not. If matched, the whole template vectors will be mapped to the raw series to compare the template vectors’ similarity with tolerance *r*.

## 3. Methodology

Our methodology involves the implementation of the fast SampEn algorithms, extracting relevant design parameters for the hardware implementation on the FPGA, and profiling the SampEn algorithms at different levels, which reveals how their performance evolves when increasing the data length.

1. **SW design**: One of the objectives of evaluating the fast SampEn algorithms is to extract the statistics needed for their hardware implementation. These statistics are used for their implementation in C/C++ programming languages toward their later evaluation when ported to the FPGA.
2. **HW design**: The implementation of the fast SampEn algorithms in C/C++ programming language facilitates their implementation on the FPGA thanks to the use of high-level synthesis (HLS) tools, such as Vivado HLS. This tool allows the translation of high-level programming language (C/C++/OpenCL) to a hardware-descriptive language (HDL) while providing estimations of latency or resource consumption among other metrics. Although Vivado HLS offers several optimizations to improve performance or area consumption, their use demands a more profound analysis and a deep design-space exploration than is intended for our algorithm’s comparison. Therefore, no hardware optimizations were used, and their evaluation is out of the scope of this work.
3. **SW profiling**: The achieved performance of the different SampEn algorithms is used as a highly efficient indicator of how the data length impacts their performance.
4. **HW profiling**: The reports from the Vivado HLS tool when converting the C/C++ implementation of SampEn algorithms into HDL are used for latency estimation. The synthesis of the HDL code using the Vivado flow provides realistic measurements of the FPGA resource, power and energy consumption.
  These metrics are used to evaluate the quality of the SampEn algorithms implemented on the FPGA.

**Figure 1:**
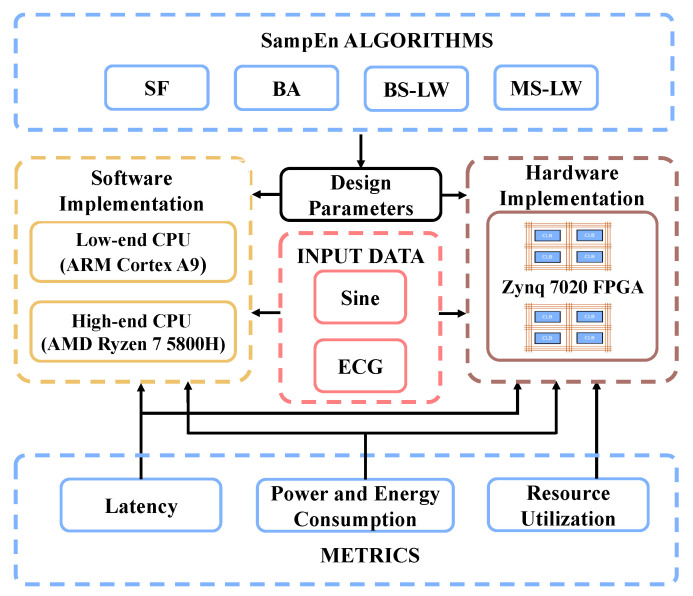
Methodology and framework of SW/HW SampEn. The data, algorithms, platforms, and metrics are involved in this work.

Figure 1 summarizes our methodological approach. On the one hand, experiments using synthetic data (sine waves) and ECG data are used to evaluate four fast SampEn algorithms implemented on three different technologies: a low-end CPU (ARM Cortex 9), a high-end CPU (AMD Ryzen 7) and the FPGA (Zynq 7020). Time latency, power and energy consumption are the metrics used in this profiling. Resource utilization of the FPGA designs are also recorded. On the other hand, design parameters for the hardware implementation on the FPGA are extracted through an analysis of the C/C++ implementation of the fast SampEn algorithms.

### 3.1. Input Data

SampEn, as well as other algorithms used for the extraction of the entropy information, has a strong dependency to the type of input data. In order to properly evaluate the impact of the characteristics of the input data over the implementation of SampEn algorithms, two different types of input data are used.

1. **Sine wave signals (sine)**: Signal sources in nature often have a periodic rhythm with noise. As input data, sinusoidal waveforms with random Gaussian noise are employed as synthetic input data. This allows the evaluation of the noise in the design parameters of the SampEn. In our experiments, the signal-to-noise ratio (SNR) of the input signals spans from 100 to 5 dB, while the length of the input data extends from 10 to 20k signals.
2. **ECG signals**: Human ECG data are a common physiological signal. To validate our studies, we use the MIT-BIH [34,35] ECG dataset. Up to 96 records are used for SampEn calculation within the same length of Sine data.

### 3.2. Metrics

Different metrics are used to profile the fast SampEn algorithms on the different technologies.

#### 3.2.1. Execution Time

The execution time of the implemented SampEn algorithm determines if a real-time response is achievable. The increment of the execution time is expected to be directly related to the data length. Nonetheless, due to the nature of the SampEn algorithms, the execution time estimation is determined by the input values. For comparison, several experiments are performed on the software version of the SampEn algorithms to retrieve the statistics needed to estimate the execution time of the FPGA implementations.

#### 3.2.2. Resource Consumption

The resource consumption of the FPGA implementations of the SampEn algorithms is a critical parameter. The demand for resources increases with the increment of the data length. However, since each algorithm has different resource demands, long data exceeding resources limitation may not be supported when implementing some SampEn algorithms on certain low-end FPGAs.

#### 3.2.3. Estimated Power and Energy Consumption

FPGAs are well known for their power efficiency. Multiple applications using SampEn must be deployed on embedded devices, which limits the power budget. The power consumption of the compared SampEn algorithms provides valuable information when selecting which algorithm runs on a power-constrained FPGA device. Similarly, the energy consumption is obtained from the power consumption and the execution time.

## 4. Extraction of Design Parameters

Even though SampEn applications already have configurable parameters, including data length (N), tolerance (r), and dimension (m), hardware architectures require extra design parameters. These design parameters are data-dependent, limit resource utilization, and can be used to estimate hardware latency.

- SF SampEn architectures need to traverse two rounds of *N* times loop body to compare template vectors and calculate their matched count.
- BA SampEn designs need to ensure buckets space distribution in advance, which preserves resources and assists in developing a fault tolerance mechanism to prevent abnormal data. Fortunately, these processes have a time complexity of one square, and their time consumption benefits the HW design by reducing the storage capacity.
- LW SampEn techniques based on sorting algorithms consist of two steps: first, sorting to make potential matched components closer together in space; and second, similarity match search with reduced unnecessary comparison. The distribution of matched ranges after sorting can be used to measure the computation latency, and the comparison, exchange, and merging times could be used to estimate the sorting module latency.

The SampEn algorithms’ software implementations (C/C++) are compared and used to extract those parameters required for their hardware implementations. With the above-mentioned design parameters, the framework of this study introduces a fast, highly efficient hardware architecture construction that depends on distinct algorithms. For our evaluation, we set parameters as m=2 and r=0.15 in both software algorithms and hardware architectures implementations, which are usually accepted in SampEn applications [4,36,37,38]. These parameters can be easily adapted to other configurations automatically. The templates’ distance defined here is the most accepted Chebyshev distance, the maximum norm between two vectors.

### 4.1. BA SampEn

**Figure 2:**
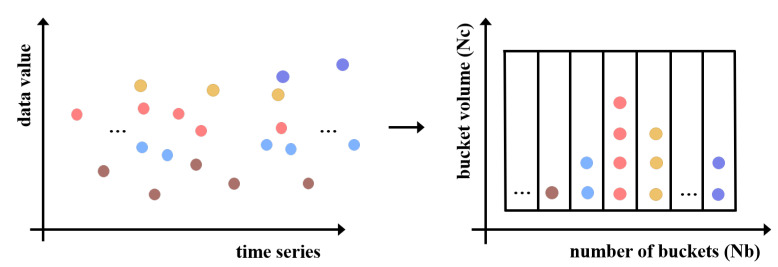
The illustration of buckets initialized by the value of elements. Similar components, we use dots in same colors to illustrate candidates in data series, are relocated to the same bucket or adjacent buckets.

BA SampEn is a memory-intensive algorithm owing to the requirement of storing candidates in buckets in advance. The graphical illustration of the BA algorithm is shown in Figure 2. Data series elements are assigned to buckets based on their value. Similar-value components are moved into the same bucket. These buckets are sorted by value in an ad hoc manner. Within tolerance *r*, neighbor bucket components are more commonly matched. As illustrated in Figure 2, the number of buckets ($N_{b}$) and their volume ($N_{c}$) are critical factors for implementing BA SampEn in hardware and determining the initialization space of buckets. The storage space for buckets is $N_{b}*N_{c}$ for candidates.

Moreover, the crucial determinants impacting the estimation of the algorithm’s delay are $N_{b}$ and $N_{c}$. As demonstrated in Table 1, these two settings also impact hardware latency. The appropriate extraction of $N_{b}$ and $N_{c}$ is critical for an accurate estimation of latency. The worst latency for BA shares the same time complexity as SF because all element is ported to a single bucket. Considering the cost in storage space remapping, SF architectures perform better than BA at this condition. However, BA has an advantage in even distribution data series, where the time complexity becomes O(N), better than other sorting algorithms. In this ideal condition, the estimated latency shown in Table 1 has excellent performance. The similarity match comparison needs the $t_{iter}$ delay. The width of $N_{nw}$ is usually 2m+1. Parameters of $r_{1},r_{2},r_{3},T_{a}$ and $T_{i}$ could be ignored for simplification. The total latency of BA could be simplified estimated as $T_{BA\;latency}=N_{b}*N_{c}*N_{c}*t_{iter}$, while the latency for LW in this process is $5*N_{n}*N*t_{c}$ in simplification. Considering *N* in LW is much bigger than $N_{b}$ in BA, BA has a great advantage in such a condition.

**Figure 3:**
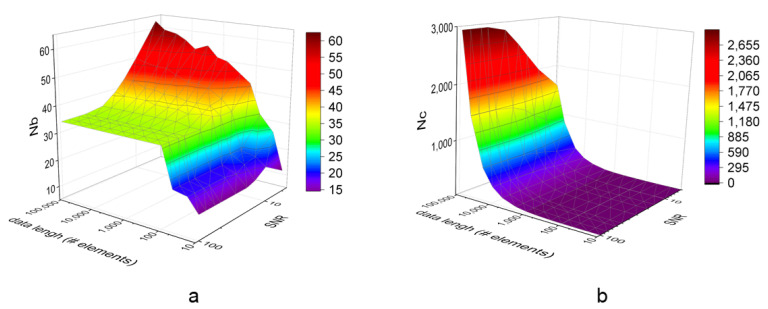
Distribution of $N_{b}$ and $N_{c}$ in BA SampEn for Sine data. (**a**) $N_{b}$ distribution increases with data length and noise but converges to long-length data. (**b**) $N_{c}$ distribution increases with data length and the $N_{C}$ volume falls with increased noise (low SNR).

**Figure 4:**
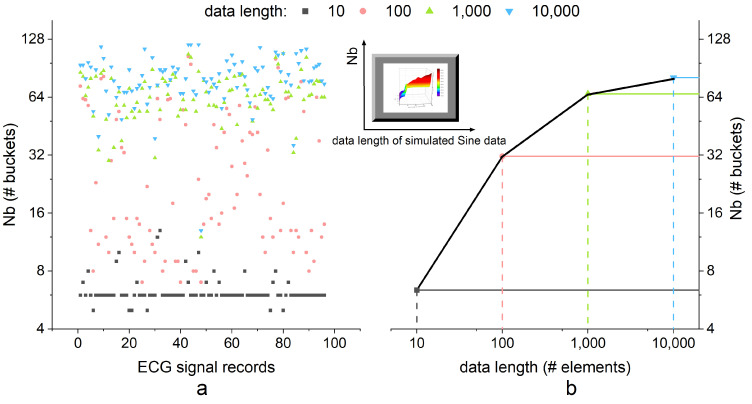
The $N_{b}$ distribution for ECG data. Nb increases with data length and reaches a boundary. (**a**) Distribution of $N_{b}$ in all ECG records. (**b**) Average distribution curve, which similarly rises and converges, same as Sine data.

The number of $N_{b}$ depend on the signal quality and length as shown in the simulated Sine data in Figure 3. Noisy signal usually has large $N_{b}$ and low $N_{c}$ for its variance. Similarly, long length data usually have big $N_{b}$. Notice that $N_{b}$ will approach a certain level regardless of data length. In experiments using Sine data, the $N_{b}$ varies from 34 to 60 in data lengths exceeding 100 elements. The $N_{b}$ rises rapidly with low SNR signals (high noise). Figure 3b also depicts the $N_{c}$ raise with the data length. Except for synthetic data, real-world ECG data also have the phenomenon that $N_{b}$ arrives at a platform with the data length increase in Figure 4. This constraint for $N_{b}$ helps hardware BA SampEn save storage space with the proper design parameter.

### 4.2. LW SampEn

**Figure 5:**
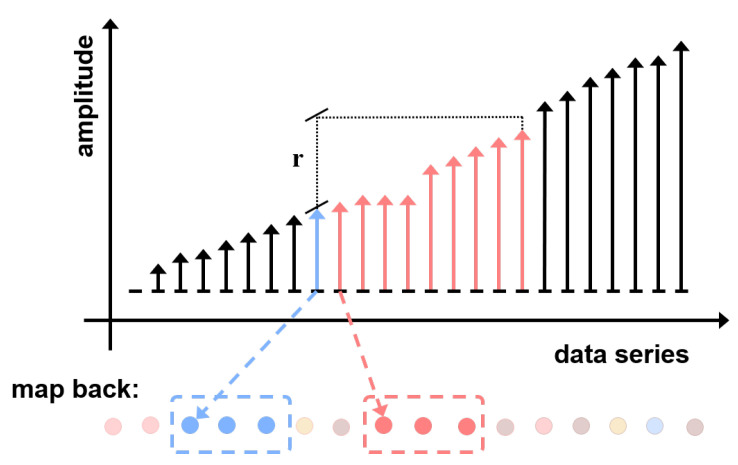
The illustration of the LW process. When transferred back to the original sequence, the sorted sequence can avoid unnecessary comparisons.

LW compare similarity and count matched number on sorted sequence. The element of sorted sequence is quickly mapped back to the raw data sequence to locate the template vectors as shown in Figure 5. Sorting is the foremostprocess of LW. BS and MS sorting algorithms are used for the benchmark.

**Figure 6:**
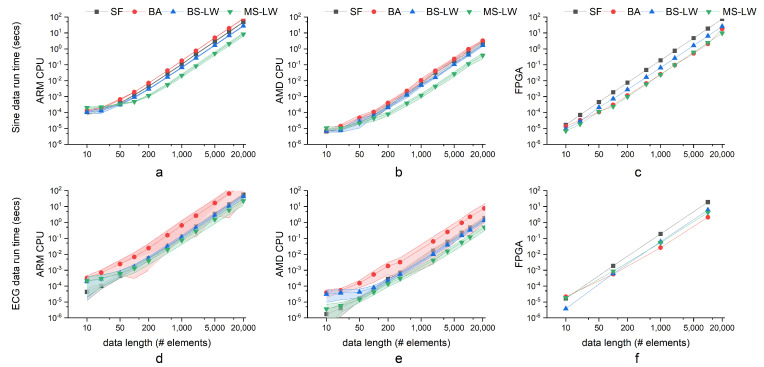
Execution time profiling. Four SampEn algorithms and two datasets are implemented on CPUs by C/C++ and FPGA. (**a**) Sine data results on the ARM CPU; (**b**) Sine data results on AMD CPU; (**c**) Sine data results on FPGA; (**d**) ECG data results on the ARM CPU; (**e**) the ECG data results on AMD CPU; (**f**) the ECG data results on FPGA. For both datasets, MS-LW is usually the fastest SampEn algorithm among all algorithms. SF is always stable with the lowest standard deviation. BA is dependent on an even distribution of data values, which results in a high standard deviation and overlap with other algorithms, especially in ECG data. Nevertheless, the best estimate of BA in hardware is state of the art. MS-LW usually has the best time performance in both CPU and FPGA environments.

BS compares two neighbor elements at first before exchanging their value and making re-locations if needed. Our experiments estimate the data exchange time in the BS process. Obviously, data exchange only occupies a tiny proportion of the total operation. The bottleneck for BS is also the unnecessary comparison. To better estimate the latency, we take both the data comparison and exchange in BS in Table 2. MS is faster, and the latency is not a bottleneck any more, especially when compared with a similarity match process. In Table 2, we could simply use the maximum limit of the MS algorithm. The results of FPGA are shown in Figure 6c,f.

In the next LW process after BS or MS, the $T_{LW}$ has three parameters of $t_{c},N_{n},N$. $t_{c}$ is the match comparison check for two template vectors, $N_{n}$ is the potential number matched within tolerance *r*, and *N* is the data length. Correlation parameters $c_{1},c_{2}$ could also be ignored here.

## 5. Experimental Results

Our evaluation of the fast SampEn algorithms is performed on different technologies. For software performance analysis with simulated Sine data and real-world physiological health ECG data, an embedded low-end CPU (ARM dual-core Cortex-A9) and a high-end CPU (AMD Ryzen 7 5800H) are employed. For the low-end CPU an embedded processor, ARM Cortex-9 running Ubuntu 18.04 is used. For the high-end CPU, a Ubuntu 20.04 subsystem is installed on Windows 10.

Regarding the FPGA implementation, a Pynq Z2 board with a Xilinx Zynq 7020 FPGA is used. This zynq XC7Z020-1CLG400C chip has Artix7 FPGA core with 280 BRAM, 220 DSP, 106,400 FF, and 53,200 LUT. The fast SampEn algorithms under evaluation implemented in C/C++ are converted to HDL language using Vivado HLS 2019.2. Parameters such as the resource and power consumption are obtained after synthesis using Vivado 2019.2.

### 5.1. Execution Time

Figure 6 depicts the execution time for data lengths ranging from 10 to 20,000 elements on a low-end CPU (ARM CPU), a high-end CPU (AMD CPU) and on the FPGA. The runtime of the SW SampEn algorithms rises significantly with the size of the input data, and MS-LW is the quickest of these fast algorithms. Since BA relies on an even distribution of data, its performance with ECG data is significantly inferior to that with Sine data. In terms of time performance, algorithms in AMD CPU are often 10 to 30 times quicker than them in ARM CPUs as shown in Table 3. Taking into consideration of a half-order-of-magnitude working frequency difference shown in Table 4, the computing efficiency of the implementation in AMD CPUs is still better than in ARM CPU. Compare Figure 6c with Figure 6f; ECG data are often sped up by 10 to 20 times, whereas Sine data are typically sped up by 10 to 30 times. The speed of BA of Sine data is usually the slowest.

The execution time on the FPGA is estimated using the equations detailed in Table 1 and Table 2 for BA SampEn and pre-sorting LW SampEn algorithms (MS-LW and BS-LW), respectively. These parameters needed can be extracted from the Vivado HLS reports. The max trip count reported in the latency section of the Vivado HLS report is replaced by the average variables obtained from estimating average latency and described in Table 1 and Table 2. The max reported trip count in the Vivado HLS report is comparable to our estimation with negligible differences. For SW SampEn, the trip count depends on the data length explicitly. Since the min and max latencies for the SW SampEn algorithm are very close, their average is used as the estimated time latency.

Figure 6c,f details the FPGA execution times estimated for each SampEn algorithm. The execution time of BA is obtained using the equations in Table 1, while BS-LW and MS-LW are obtained from the equations in Table 2. The estimations depicted in Figure 6c,f show that BA SampEn in the best condition presents similar execution times with pre-sorting LW SampEn algorithms for data lengths ranging from 100 to 10k elements. Thus, while MS-LW is the fastest algorithm for most data lengths, BS-LW is significantly slower. Compared to their respective software implementation, the execution times on hardware FPGA show that BA SampEn can perform comparably with some pre-sorting LW SampEn algorithms in their best condition. It is because BA relies on the distribution of data and their distribution of buckets. The worst condition of BA is that all elements are ported to a single bucket and make it perform like SF. The software simulation in Figure 6 also proves this analysis.

With the understanding by analysis and experiment, the time character of fast SampEn helps an early selection of the SampEn algorithms when looking for real-world implementation. A design flow and corresponding SW/HW tools make quick SampEn implementation possible by analyzing this research framework.

### 5.2. Resource Consumption

**Figure 7:**
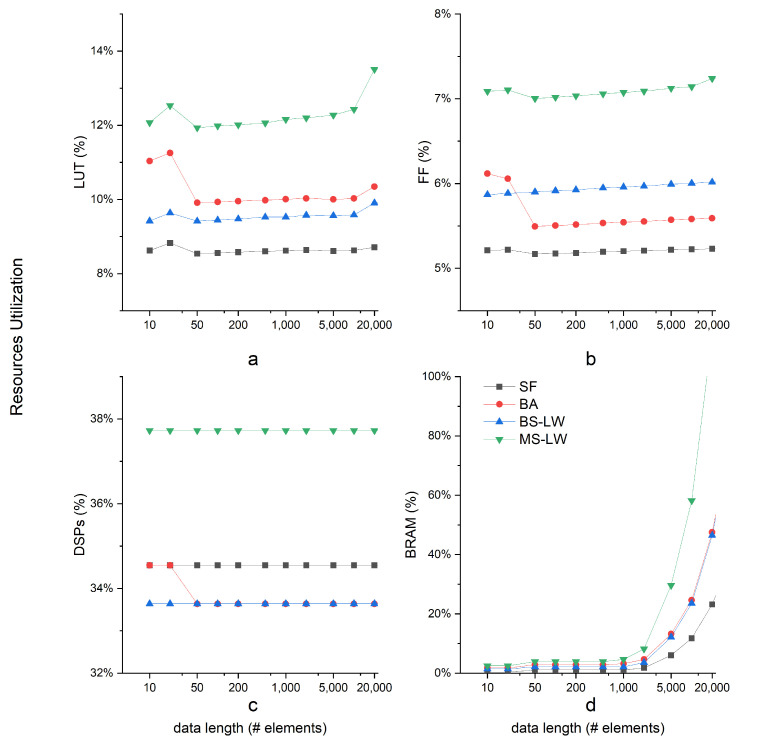
Resource consumption after-synthesis within a Xilinx Zynq 7020. Storage (BRAM) resources grow substantially with data length.

Figure 7 details the resource consumption of each SampEn algorithm obtained after Vivado synthesis. The consumption of LUTs, FFs, SRLs or DSPs remains approximately constant when increasing the data length. Due to the characteristics of SampEn, the limiting resource are the BRAMs. Internal memory for intermediate storage when performing the template matching is directly related to the data length. Since up to 280 BRAMs can be consumed in the Xilinx Zynq 7020, data lengths ranging from 10k to 100k elements determine what SampEn algorithms can be allocated.

Sort-based SampEn solutions present a higher BRAM consumption due to the internal memory needed for the sorting algorithms. MS-LW is the algorithm that presents a higher demand for internal storage, mainly because the additional memories needed for the MergeSort algorithm. BA SampEn presents a similar resource consumption to BS-LW, requiring more memory resources for the buckets storing high probability matched candidates. It is motivated because the candidates’ number in a bucket depends on the input data. To minimize this dependency, we used the number of data lengths for redundancy, which makes the bucket size need large storage space.

These resource consumptions reveal the maximum data length supported for each algorithm, which can be a limiting factor for certain applications.

### 5.3. Power and Energy Consumption

**Figure 8:**
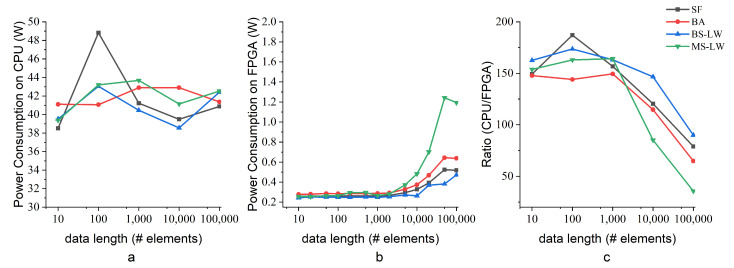
Comparison of power consumption for Sine data. (**a**) Tests on AMD CPU consume a great deal of power regardless of data length; (**b**) implementations on the FPGA consume less power than equivalent implementations on the AMD CPU but the power consumption increase with the data length increases; (**c**) the power consumption ratio of AMD CPU and Zynq 7020 FPGA, which decreases when the data length increases.

Figure 8 shows the evolution of the power consumption of the SampEn algorithms running on the AMD CPU and on the FPGA when increasing the data length. Figure 8a is the power test for SampEn algorithms on AMD CPU. A smart charger of “MIJIA” records the power consumption. The power consumption is recorded by multi times on a high-performance personal computer (PC). The static power, which is the offset power consumption due to the OS when the algorithm is not running, rounds to 21 W. When the PC continuously runs SampEn algorithms on the AMD CPU, the stable power numbers range from 38 W to 44 W. Figure 8b is the HW power consumption on FPGA. Although it is not depicted, the power consumption of all SampEn algorithms is dominated by the dynamic power consumption, which ranges from an initial up to 57% to 87% of the total power consumption when increasing the data length. Nonetheless, the power consumption of the SampEn algorithms running on the AMD CPU is significantly higher than on the FPGA. Figure 8c compares power consumption between AMD CPU and FPGA. The AMD CPU consumes several orders of magnitude more than FPGAs. However, the power consumption of the FPGA SampEn implementations increase with the data length because of the increased memory resource consumption.

There is a significant difference between the power consumption of the SampEn algorithms under evaluation implemented on the FPGA. The sorting algorithm presents a high impact on the estimation of the power consumption, showing that BS-LW SampEn and MS-LW SampEn are the lowest and the highest power demanding algorithms, respectively. The growth of MS-LW SampEn in power demands can be related to its resource consumption, especially BRAM, which is motivated by multiple internal memory operations.

**Figure 9:**
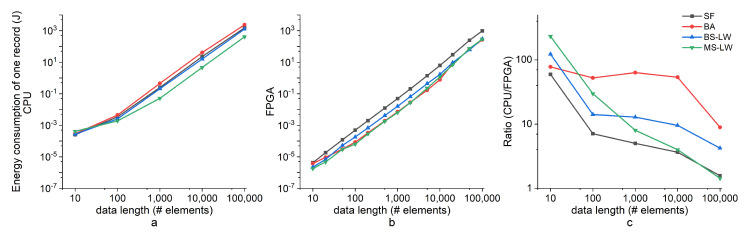
Comparison of energy consumption between an FPGA implementation and the versions running on an AMD CPU performed for a single Sine data sample. (**a**) The energy consumption of the AMD CPU; (**b**) the energy consumption the Xilinx Zynq 7020 FPGA; (**c**) the energy efficiency ratio of the AMD CPU and FPGA. The power consumption of SampEn algorithms on the FPGA is more efficient but rises to close to that of SampEn algorithms on the AMD CPU in long-length data.

Figure 9 shows the result of energy (in J) comparison for a single record. Notice that the implementation of SampEn on FPGA is much more power efficient, performing several orders of magnitude better than the AMD CPU for short input data lengths. Nonetheless, its power efficiency decreases with the increment of the input data length because large input data lengths increase the resource utilization of the hardware architecture, leading to a high power consumption.

## 6. Discussion

Our evaluation of promising fast SampEn algorithms on different technologies leads to interesting results. The implementations of fast SampEn algorithms on the FPGA showed a significantly higher power efficiency while providing a similar performance, compared to running on a high-end AMD CPU. Nonetheless, higher performance can be achieved on the FPGA by exploiting the optimizations available on the HLS tool used to generated the FPGA designs.

For the evaluated SampEn algorithms, the BA SampEn performance has a higher dependency with the data distribution. In the worst condition, BA SampEn has a $N^{2}$ time complexity, like SF SampEn. However, when the input data are evenly distributed, BA performs similar to the MS-LW algorithm. MS-LW performs better when implemented on the AMD CPU and on the FPGA in terms of time latency and power consumption. It comes from the fact that MS-LW saves time in data space remapping and avoids most unnecessary match comparisons, especially in the first element comparison of template vectors.

In the large data analysis, as shown in Figure 6, both Sine wave (Sine) data and ECG data share similar latency within the same methods, while SF SampEn is simple and has a stable execution time regardless of being on the AMD CPU or on the FPGA.

As far as we know, in some related work in Table 4, fast SampEn is a concern in research and application. However, implementation of SampEn on the FPGA lacks research. In this work, we verify that fast SampEn algorithms implemented on FPGAs offer considerable advantages in speed, especially in power efficiency. However, there is a need for a deeper exploration of the design parameters when porting SampEn algorithms to FPGAs, and the close dependency of the achievable performance and design architecture with the characteristics of the input data.

Finally, through this work, we have proposed a methodology for the analysis of SampEn algorithms and their implementation on reconfigurable architectures, such as FPGAs.

## 7. Conclusions

Promising fast SampEn algorithms, such as bucket-assisted (BA) or pre-sorted lightweight (LW) algorithms are profiled in this research by synthetic and real-world ECG input data. Those algorithms were evaluated on different computational technologies such a high-end CPU and on an FPGA in terms of performance, resource, power and energy consumption. The FPGA implementations of fast SampEn demonstrates to be several orders of magnitude (two order for 20,000 input data) more power efficient than an equivalent implementation on a AMD CPU, in addition to offering similar performance. Overall, MS-LW performs better in both technologies when compared to other LW-based SampEn algorithm or to BA SampEn. Nonetheless, a deep study and analysis of the fast SampEn algorithms under evaluation is needed in order to retrieve design parameters toward their implementation on an FPGA.

## Figures and Tables

**Table 1:** BA SampEn hardware latency estimate.

Parameter	Time Performance Estimation
N	# elements, length of the input sequence
$\;N_{b}$	# buckets
$\;N_{c}$	# candidates in a bucket
$\;N_{nw}$	# corrected neighbor buckets for similarity comparison, usually is 2m+1
$\;r_{1},r_{2},r_{3}$	correction parameters, could be ignored for simplicity
$\;t_{iter}$	Latency for one time similarity comparison
$\;T_{i}$	time for initiation, could be ignored for simplicity
$\;T_{a}$	latency for bucket assignment, could be ignored for simplicity
$\;t_{1}$	$=t_{iter}*N_{c}+r_{1}$, latency for one template vector compared with one bucket
$\;t_{2}$	$=t_{1}*N_{c}+r_{2}$, latency for comparisons between two buckets
$\;t_{3}$	$=t_{2}*N_{nw}+r_{3}$, latency for comparisons between one bucket and its neighbors
$\;T_{BA\;latency}$	$=N_{b}*t_{3}+T_{i}+T_{a}$, latency clocks for BA
$\;t_{BA\;delay}$	$=T_{BA\;latency}/freq$, execution time for BA

**Table 2:** SampEn hardware latency estimate for BS-LW and MS-LW.

Parameter	Time Performance Estimation
	BubbleSort (BS)
$\;t_{comparison}$	latency for a comparison in bubble sorting
$\;t_{exchange}$	latency for a exchange in bubble sorting
$\;r_{1}$	exchange times count in bubble sorting
$\;r_{2}$	extra latency in each iteration of bubble process
$\;t_{1}$	$=t_{comparison}*\sum_{i=1}^{N}i$, latency for comparison operation in Sorting
$\;t_{2}$	$=t_{exchange}*r_{1}$, latency for exchange operation in Sorting
$\;t_{3}$	$=r_{2}*\left(N-1\right)$, could be ignored for simplicity
$\;T_{BS\;latency}$	$=t_{1}+t_{2}+t_{3}$, latency clocks in bubble sorting
	MergeSort (MS)
$\;M_{l}$	= ceil(log2(N)), number of layers for merge operation
$\;N_{m}$	$1,2,...M_{1}$
$\;M_{i}$	= $ceil(N/\left(2^{N_{m}}\right))$, number of subsequence to be merged in a layer
$\;t_{m}$	latency for an operation of element merge
$\;t_{s}$	latency for merging two subsequence
$\;t_{l}$	latency for merging in a layer
$\;t_{1}$	$=t_{m}*N*M_{l}$, total latency in merging process
$\;t_{2}$	$=t_{s}*\sum_{N_{m}=1}^{M_{l}}ceil(N/\left(2^{N_{m}}\right))$, total latency for operations between subseries
$\;t_{3}$	$=t_{l}*M_{l}$, total latency for operations between layers
$\;T_{MS\;latency}$	$=t_{1}+t_{2}+t_{3}$, latency clocks in merge fast sorting
	LW SampEn
N	# elements, length of the input sequence
$\;N_{n}$	# constant element match within *r* in sorted sequence
$\;c_{1}$	latency in operation for one time constant similarity search comparison
$\;c_{2}$	latency of memory operations
$\;t_{c}$	time for similarity comparison check
$\;T_{LW}$	$=\left(t_{c}*N_{n}+c_{1}\right)*N+c_{2}*N$, latency for similarity search in sorted series
$\;T_{BS-LW}$	$=T_{LW}+T_{BS\;latency}$, latency clocks in BS-LW
$\;T_{MS-LW}$	$=T_{LW}+T_{MS\;latency}$, latency clocks in MS-LW
$\;T_{*S-LW\;latency}$	$=T_{BS-LW}\;or\;T_{MS-LW},$
	dependingonthesortingalgorithm
$\;t_{*S-LW\;time}$	$=T_{*S-LW\;latency}/freq$, execution time for BA-LW or MS-LW

**Table 3:** Speedup of CPUs and FPGA implementations. AMD CPU and FPGA speedup calculations are compared with ARM CPU implementations as a reference. The value in the table has a format of mean ± std for all data lengths in experiments.

Methods	Sine Data			ECG Data		
	ARM	AMD	FPGA	ARM	AMD	FPGA
SF	1.0 ± 0.0	17.5 ± 2.5	1.3 ± 1.7	1.0 ± 0.0	25.8 ± 3.0	1.3 ± 0.1
BA	0.6 ± 0.1	18.4 ± 2.9	7.2 ± 1.7	0.2 ± 0.0	12.3 ± 1.4	0.1 ± 0.0
BS-LW	1.5 ± 0.2	15.2 ± 1.5	2.4 ± 3.1	1.1 ± 0.3	25.3 ± 8.0	0.5 ± 0.1
MS-LW	4.1 ± 1.9	17.4 ± 3.1	4.7 ± 8.1	1.7 ± 0.7	32.1 ± 6.6	0.7 ± 0.0

**Table 4:** Fast SampEn related works. The SW fast SampEn on CPUs performs well; the HW SampEn on FPGA, even when run at a low frequency, is comparable to other works.

Parameter	Work1 [21]	Work2 [39]	Work3 [24]	This Work		
	SW	SW	SW	SW	SW	HW
Processor	Intel(R) Core(TM) i5-4590	Intel TM i7-8550U	Intel Xeon E5-1620	ARM Cortex-A9	AMD Ryzen 7 5800H	Artix 7 FPGA
Frequency	3.3 GHz	1.80 GHz	3.6 GHz	650 MHz	3.2 GHz	100 MHz
Data	EEG	RR interval	RR interval	Sine, ECG	Sine, ECG	Sine, ECG
Data length	256	30	50k	50	100k	20k
Algorithm	Fast SampEn	SampEn	Fast SampEn	SampEn, Fast SampEn	SampEn, Fast SampEn	SampEn, Fast SampEn
Best latency Power	11.07 ms	0.24 ms	500 ms	0.33 ms	9.79 s	9.71 s
	-	-	-	-	42.5 W	0.58 W

## Data Availability

The data is available under request.
